# Editorial: Delivery of Locally-Acting Agents to Intracellular Targets

**DOI:** 10.3389/fphar.2020.593064

**Published:** 2020-09-23

**Authors:** Andrey A. Rosenkranz, Salvatore Salomone, Alexander S. Sobolev

**Affiliations:** ^1^Laboratory of Molecular Genetics of Intracellular Transport, Institute of Gene Biology, Russian Academy of Sciences, Moscow, Russia; ^2^Department of Biophysics, Faculty of Biology, Lomonosov Moscow State University, Moscow, Russia; ^3^Department of Biomedical and Biotechnological Sciences, University of Catania, Catania, Italy

**Keywords:** aptamers, membrane trafficking, modular nanotransporters, anticancer drugs, Auger electron emitters, poly(ADP-ribose)polymerase inhibitors, anti-tuberculosis agents, intracellular targets

The vast majority of pharmaceuticals penetrate into tissues and cells regardless of their actual requirements for treatment. This leads to side effects, which restrict the use of drugs and require decrease of the treatment doses. In addition, many of potential pharmaceuticals cannot be used due to poor cell permeation, because their charge or large size limit their penetration through biological membranes. For these reasons, subcellular drug delivery became a rapidly growing area of research in the medical and pharmaceutical field. Many biologically active agents can be transported into a specific cell compartment in order to exert their activity or to achieve higher activity. There are drugs, like photosensitizers (Rosenkranz et al., 2000), radionuclides emitting short-range particles (Sobolev, 2018; Rosenkranz et al., 2020), anticancer, antimicrobial, and antiviral drugs (Torchilin, 2014), that can exert their maximum effect within a certain compartment. In spite of considerable progress in development of subcellular delivery approaches, many types of biologically active molecules, potentially exploitable in clinical settings, are on the waiting list. Special interest is attracted by approaches to make macromolecules, like antibodies (Slastnikova et al., 2018), aptamers (Marshall and Wagstaff) or natural regulatory proteins, e.g., transcription factors (Ulasov et al., 2018) cell-penetrable and capable to specifically interact with subcellular target molecules of choice within the target cells. All the above agents could be named as locally-acting ones, because their actions or interactions are limited to specific subcellular compartments; they also might need special delivering vehicles and can be employed for cell-specific impact. The main goal of this Research Topic is to highlight the current state of delivery vehicles for locally-acting drugs into target compartments of particular cells. Some previous achievements in this field were discussed in the Research Topic “Targeted Subcellular Delivery of Anti-Cancer Agents” published in “Frontiers in Pharmacology” (2018-2019). The most recent findings and reviews of new ideas to show the way forward in developing strategies to efficiently deliver drugs to specific subcellular sites of target cells are presented in this Research Topic. Intracellular membrane trafficking pathways, which promote the transport of the active molecule into the subcellular locations, have a fundamental importance for design of subcellular targeting. This issue is discussed in detail in the review article by Kumar and colleagues (Kumar et al.). Another task of intracellular targeted delivery for treatment of a number of diseases, particularly cancer, is design of highly specific molecular targeting. DNA aptamer molecules are a rapidly growing tool in this field that can be applied for specific cell surface targeting, subsequent internalization and interaction with intracellular target molecules (Marshall and Wagstaff). Currently, aptamers have a few limitations to overcome before they can be broadly accepted for therapy. One of them is determination of specific targets of the selected aptamers. Another one is insufficient endosomal escape that restricts access of aptamers to many intracellular targets. Nevertheless, the use of aptamers as components of multifunctional delivery systems has already broad prospects.

The approach based on the use of recombinant polypeptides makes possible combining several transport functions in a single carrier molecule. Such constructs, called modular nanotransporters (MNT), possess not only the ability to recognize target cells and enter cells, but also to escape from endosomes and be actively transported into the nucleus. Karyagina and co-workers developed new transport vehicles for targeting the nuclei of cancer cells with overexpression of epidermal growth factor receptor (EGFR). The possibility of using internalizable anti-EGFR antibody mimetic as an EGFR-targeting ligand for drug transport into the cancer cell nuclei was examined (Karyagina et al.). This resulted in enhanced cytotoxicity of Auger electron emitter^111^ as a result of its attachment to this new MNT.

Poly(ADP-ribose)polymerase-1 (PARP1), a DNA repair enzyme highly expressed in the nuclei of mammalian cells, is an example of an important nuclear target. PARP inhibitors have given rise to promising new cancer therapies and treatment strategies. The PARP inhibitors also can act as radiosensitizing agents by delaying single strand breaks repair and causing hardly repairable double strand breaks. The review of Jannetti and co-authors uncovers the utilization of these inhibitors in combination therapies and in scaffolds for imaging agents, radiotherapeutics, and radiotheranostics (Jannetti et al.).

The rise in incidence of multidrug-resistant and extensively drug-resistant tuberculosis necessitates the search for new drugs for treatment of this dangerous infectious disease caused by the intracellular bacilli. Better tissue distribution is highly desirable for these candidate drugs. Therefore, the article of Tanner and co-workers is devoted to study organ distribution for two new promising drugs for tuberculosis treatment (Tanner et al.).

## Author Contributions

All authors listed have made a substantial, direct, and intellectual contribution to the work, and approved it for publication.

## Funding

AR and AS are indebted to the funding support of the Russian Science Foundation (grant #17-14-01304).

## Conflict of Interest

The authors declare that the research was conducted in the absence of any commercial or financial relationships that could be construed as a potential conflict of interest.
